# A Highly Rare Cause of Lumbar Spondylodiscitis with Epidural Abscess: *Actinomyces israelii*

**DOI:** 10.1155/2014/469075

**Published:** 2014-06-12

**Authors:** Mahir Kapmaz, İsmail Gülşen, Naciye Kış, Seniha Başaran, Lütfiye Öksüz, Nezahat Gürler

**Affiliations:** ^1^Bitlis State Hospital, Infectious Diseases and Clinical Microbiology Department, Bitlis, Turkey; ^2^Bitlis State Hospital, Neurosurgery Department, Bitlis, Turkey; ^3^Bitlis State Hospital, Radiology Department, Bitlis, Turkey; ^4^Infectious Diseases and Clinical Microbiology Department, Istanbul University, Istanbul Medical Faculty, Istanbul, Turkey; ^5^Department of Medical Microbiology, Istanbul University, Istanbul Medical Faculty, Istanbul, Turkey

## Abstract

*Actinomyces* species may lead to slowly progressive infection of almost any site once mucosal breakdown exists; hence, it has the name “great pretender.” Its diagnosis may be unthinkable unless proper cultures/histologies are taken. We describe a patient with lumbar spondylodiscitis and epidural abscess. This is an exceptional another disease by actinomycosis.

## 1. Introduction

*Actinomyces *species are the flora members of oropharynx, colon, and vagina, but mucosal breakdown may lead to slow progressive infection of almost any site [1]. Being the most common oral-cervicofacial disease, thoracic, abdominopelvic, and less commonly central nervous system and musculoskeletal involvement are seen. Because of its wide variety of clinical presentations such as tongue involvement, acute cholecytitis, and inferior vena cava syndrome actinomycosis was once given the name “great pretender” [2, 3].

Actinomycosis of the thoracic spine with spinal cord compression is uncommon though well described [4–6]. Its diagnosis may be unthinkable unless proper cultures and/or histology are taken. Lumbar spinal involvement is more scarce than thoracic disease [7].

We describe a patient with lumbar spondylodiscitis and epidural abscess, who presented initially with pneumonia and later developed low back pain with paraparesia. This is an exceptional another disease by actinomycosis.

## 2. Case Report

An 82-year-old male was admitted to our clinic with low back pain. From presenting medical history, he was admitted to a tertiary center with fever, cough, and weakness twenty days ago. There, he was in stupor position with right posteriobasal consolidation with bilateral low posterior pleural thickening and postobstructive renal failure due to urolithiasis. Following hospitalisation with a diagnosis of community-acquired pneumonia and acute renal failure, his renal failure resolved with urethral catheterisation and antibiotherapy. His new beginning symptom of backache was associated with his prostate carcinoma history and a probable metastasis. A PET-CT was planned, and he was discharged. Later, a lumbar MRI was taken due to progression of pain, which revealed L4-5 spondylodiscitis. He was hospitalized to our clinic.

**Figure 1:**
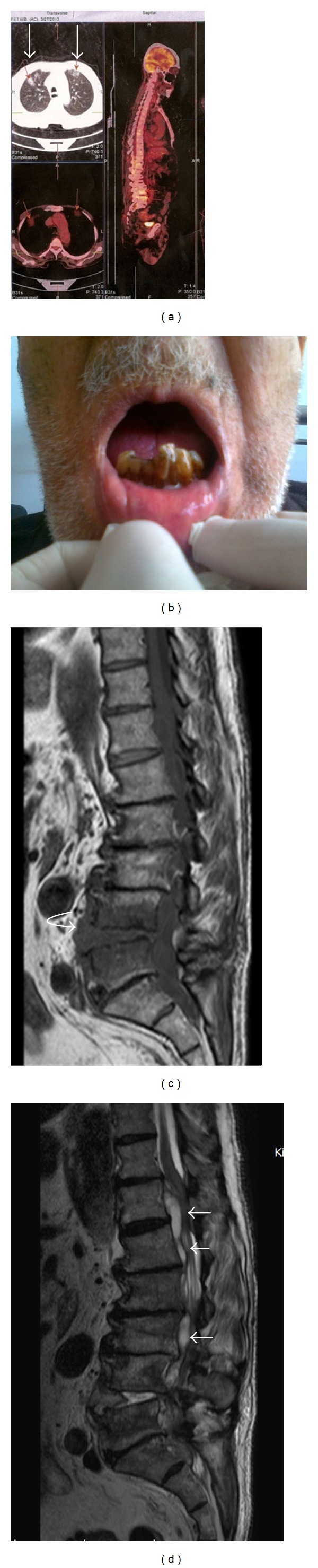
(a) PET-CT image of the patient showing pulmonary and lumbar vertebral involvement (arrows). (b) Poor orodental hygiene of the patient of heavy smoking hand-rolled tobacco. (c) T1 weighed sagittal MRI of the spine showing edema and postcontrast involvement in L4-5 vertebral bodies and postcontrast uptake L4-5 intervertebral disc (arrow) and adjacent iliopsoas muscle (not shown in this image). (d) T2 weighed sagittal MRI reveals epidural fluid with an 8 mm width at most between T12 and S1 vertebrae, in an unconnected extension with peripheral contrast involvement. There are a marked spinal cord and neural foraminal compression (arrows).* Actinomyces israelii *was isolated from the culture of the abscess and disc material.

On his personal history, he had prostat carcinoma since 2012. The pathology was adenocarcinoma Gleason score 7. He had TUR-P with goserelin every three months. The abnormal FDG uptake in pelvis which was reported on the previous one was normalised on the new PET-CT. A new abnormal uptake, however, was reported in the T6-7, T10-12 and L3-5 vertebrae and in the lungs with a comment of metastasis or pneumonia (Figure 1(a)).

On physical examination, he was on the wheelchair, and cooperated,with a fever of 37.3°C, pulse of 90/min, and blood pressure of 140/80 mm/Hg. He had poor orodental hygiene (Figure 1(b)) with normal systemic findings. His bilateral lower muscle strength was 3/5.

Laboratory revealed an ESR of 80 mm/hour, CRP of 58.9 mg/dL, and WBC of 10.850/microliter. His urinanalysis revealed abundant leukocytes and erythrocytes. Multiple blood cultures were negative with no vegetation on transthoracic ECHO. In two consecutive urine cultures, carbapenem-intermediate resistant* K. pneumoniae *was isolated. 14 days of imipenem were given for complicated urinary tract infection with contact precautions. His control urine culture was negative. Tumor markers including PSA were in normal ranges. Abdomen CT revealed bilateral urolithiasis and focal sclerotic areas in pelvic bones. Brucella agglutination test was negative. The agglutination test with Coombs was taken because of its endemicity. It was positive in a 1/80 titer. With a diagnosis of brucellosis discitis, the combination of doxycycline and rifampin was added to imipenem. Streptomycin was avoided due to nephrotoxicity. After imipenem, oral ciprofloxacin was initiated as the third agent.

A new lumbar MRI taken because of nearly no regression of pain revealed L4-5 spondylodiscitis and epidural abscess (Figures 1(c)-1(d)). He underwent an urgent decompression surgery: total laminectomy, abscess drainage, and stabilisation. Contrary to our pyogenic abscess expectation, an opaque thick film tissue of rubber consistency was curetted. The culture resulted in isolation of Gram-positive branching bacteria. The identification resulted in* Actinomyces israelii*. With a diagnosis of actinomycosis spondylodiscitis, 6 weeks of crystallized penicillin 15 million IU/day were given. Oral amoxicillin 500 mg × 3/day (adjusted renal dosage) of one year was planned. Because the diagnosis of brucellosis discitis was dismissed, antibrucellosis therapy was stopped at the 6th week. ESR was regressed to mostly 55 mm/hour. At the 6th month control, he can walk with the help of a walking stick with low to moderate mechanical back pain. He has occasional urine culture positivity of* K. pneumoniae* with dysuria, and he is planned to have operation for urolithiasis.

## 3. Materials and Methods

The Gram stain revealed moderate polymorphnuclear leukocytes and rare Gram-positive branching rods. The abscess was inoculated onto 5% sheep-blood agar, Schaedler agar, and broth (BioMérieux, France). Gram-positive bacilli were recovered after 48-hour incubation.

The colonies were very firm and notably adherent to the agar surface. They had R-type white opaque colony morphology of dome shape. A negative catalase and pigment test with no formation of indole helped us to differentiate* Actinomyces* from* Propionibacterium *species. The bacteria were identified as* Actinomyces israelii* (98.2% confidence level) by the API 20A system (BioMérieux, France). The bacteria were found to be susceptible to penicillin by *E*-test according to recommendations of CLSI. The histologic examination did not reveal any clusters of the microorganism. His informed consent was taken.

## 4. Discussion

Actinomycosis is a rare, chronic, and slowly progressive granulomatous disease caused by filamentous Gram-positive anaerobic/microaerophilic bacteria from the Actinomycetaceae family [8]. It is mainly an endogenous infection and never been cultured from nature. There are no documented cases of person-to-person transmission [1]. The clinician, therefore, should avoid the interpretation of positive cultures of* Actinomyces* sp. as laboratory contaminants.

Mostly, local extension, hematogenous dissemination, and foreign bodies (such as intrauterine devices in pelvic disease) contribute to the pathogenesis. A mass characteristically enlarges across tissue planes, which may result in formation of sinus tracts that can spontaneously heal and recur [8]. It is often, therefore, misdiagnosed as malignancy and other infections, such as tuberculosis, clinically and radiologically. Due to the slow progression of the disease, both vertebral body destruction and new bone formation occur, resulting in a mottled, saw-toothed, or honeycombed appearance on X-ray. The transverse processes are also involved, in contrast to their sparing with tuberculosis [1]. Thoracic infection occurs as an extension of cervicofacial infection into the chest and mediastinal region usually as a result of aspiration of oropharyngeal content or by the spreading of abdomino/pelvic infection retroperitoneally or transdiaphragmatically. Since concomitant pneumonia exists in our patient, the infection is supposed to extend from lung (plevral thickening) to the vertebral bodies. Besides there is FDG uptake on T6-7, T10-12 vertebrae; however, the extension of infection causing epidural abscess in the lumbar vertebrae seems to be a highly rare entity. Lumbar spine is reported less to be affected [7]. Since Cope stated that the intervertebral discs are exceptionally affected in actinomycosis in 1951 [4], there are few cases of reported discitis: the first case relies on DNA analysis of the disc biopsy in a 74 years old man; the second case is secondary to a lumbar disc surgery, and the third case is as spondylodiscitis due to* Actinomyces meyeri *[9–11]. Brucellosis is very well known cause for spondilodiscitis [12]. Though a SAT titer of 1 : 160 is considered to be indicative of active brucellosis, it cannot be excluded in patients with titers lower than 1 : 160 [13]. The concomitant brucellosis seropositivity (even a result of Coombs SAT 1 : 80) confronted the picture in our case. It was supposed to be associated with the endemicity in this part of Turkey. The patient had no history of brucellosis treatment before. In an inevitable attempt, brucellosis therapy was initiated to the distressed patient who had ongoing paraparesia. Since the culture is revealing no* Brucella* sp., but* Actinomyces israelii* is more reliable, brucellosis therapy was stopped in six weeks. This is, however, probably the first case of spondylodiscitis due to actinomycosis with a concomitant low seropositivity of brucellosis.

The conditions such as poor oral hygiene, diabetes, alcoholism, HIV infection, steroid and bisphosponates use, transplantation, local tissue damage caused by trauma, recent surgery, irradiation, and male gender of mid-decades are thought to predispose individuals to the development of actinomycosis [5, 8]. The poor oral hygiene of our patient who smoked hand-rolled tobacco for 60 years and his cancer status possibly made him vulnerable to the infection. No tumor cells were observed in his pathology, which excludes the probability of prostatic adenocarcinoma metastasis.

Histological diagnosis of actinomycosis is difficult, because many specimens contain only a few granules [14]. The absence of sulfur granules from any lesion, however, does not exclude the diagnosis of actinomycosis; actinomycotic cerebral lesions have lacked histological diagnosis although culture-proven [14, 15]. Despite the fact that thorough examination was done, no microorganisms were detected in histological material of our case.

*Actinomyces *species are susceptible in vitro to several antimicrobials including penicillin G, tetracyclines, erythromycin, clindamycin, imipenem, streptomycin, and the cephalosporins [14]. Prolonged therapy with high doses especially of penicillin is crucial. Surgery must be assessed on an individual basis and may be unevitable if there are extensive necrotic tissue, sinus tracts, failure with medical therapy, unsuccesful drainage of abscesses or empyemas, and need of curettage of bone [8, 14].

As a result, our patient would be treated as brucellar spondylodiscitis if no tissue culture had been taken. Even in nontertiary centers, biopsy should be encouraged in spondylodiscitis cases. Pneumonia preceding spinal infection, with especially a poor dental hygiene, can make the desperate clinician think of actinomycosis in the differential. Maintaining good oral hygiene in the elderly is an important preventive measure for actinomycosis infections.
